# Discoloration of Gold Fillings

**Published:** 1885-12

**Authors:** S. B. Palmer

**Affiliations:** Syracause, N. Y.


ARTICLE V.

### DISCOLORATION OF GOLD-FILLINGS.

BY S. B. PALMER, D. D. S., SYRACUSE, N. Y.

I shall detain you but a very few moments. We often see articles in our journals touching upon the subject of the discoloration of gold-fillings, which present some facts and many theories. It is not my purpose to discuss in detail all these theories, but simply such as I believe have not been generally presented. It is desirable that this subject should be better understood and the facts recorded for further reference.

What are the facts? In regard to discolored fillings: First, they vary from copper color to black; second, two fillings from the same gold and in the same mouth, discolor differently; third, sometimes nearly all are black; fourth sometimes they discolor only at the cervical border near the gums.

The most frequent changes occur with one or two fillings in the same mouth. This can readily be accounted for from the fact of the use of the burnisher upon the gold when the filling has been ground down by corundum sand or pumice, which leaves upon the gold a deposit like diamond dust which cuts the instrument. Where a filling is exposed to the action of the brush this will not remain long. Figures have been given us, but we know there is not enough wearing away from the instrument to account for other known conditions.

Mercury has been set forth as a quite prominent cause of the discoloration of gold fillings, and it would seem true when in close proximity to gold. When we patch gold fillings with amalgam or put gold upon amalgam where there is a distinctly defined line, we seldom see the mercury leave the amalgams and pass over to the gold, but the gold seems stained a brownish color, so we cannot account thus for this general discoloration.

For many years I have been satisfied in my own mind as to the cause, and I have made a series of experiments which will authenticate the position taken, Lead, zinc, mercury, copper and iron will all deposit on gold, but I regard iron to exceed, by far, all the others, both in its frequency of being present and its readiness of attachment by galvanic action.

Experiment 1. An eight-penny nail was placed in a tumbler of water over night when it was removed and number twenty gold foil introduced. This remained in the water for fifteen to twenty hours and on annealing gave a decided copper color. I placed a carpet tack in water over night but without any effect whatever.

Experiment 2. A rope of gold prepared as for filling a tooth was made to receive at one end the point of a carpet tack; the rope was then bent in a loop and the two ends immersed over night in pure water. The gold was thus polarized and the iron transferred to and deposited upon the gold.

Experiment 3. When water containing zinc, tin, copper or iron was used, it coated the surface of pure gold, either in water, acid, or alkali. An amalgam filling which had been worn in the mouth was drilled into and suspended in water, and connected with small pieces of number twenty gold foil. The color under these circumstances was darker than when iron simply was used, but some was not perceptible until submitted to the influence of sulphuretted water, or sulphuretted hydrogen gas, and this was generally driven off by annealing, except when iron was sufficient in quantity to remain upon the gold.

Iron I consider the most frequent cause of this discoloration. Years ago, before the advent of annealed ware, I first tried the experiments and became convinced that the discoloration was often caused by the use of iron vessels used in cooking. It was my custom whenever I saw a discolored filling, to ask the patient what kind of acid food he or she used, and it was generally cabbage or some other vegtable cooked in an iron vessel. Now this would take off sufficient iron to show the trace upon the fllling. Iron is very often used for tanks, and if you take the pains to ask the patient, you will find that iron has frequently been derived from water stored in such tanks or conveyed in iron pipe.

Iron is also frequently given as medicine, tonics for example, and a little inquiry of your patients will show that iron has been taken in that way. Now, do not blame the physician, for iron it would seem must be taken; and then, I am pretty well convinced that this effect is a secondary one, and held in the secretions and like lime deposited upon the teeth. I regard iron as deposited by the same process. under conditions favorable to cohesive attraction.

There is one principal in nature, which if we understand, will clear up this whole subject, and that is electrical induction or polarity; for example, the atmosphere is positive, and the surface of the earth is negative. If we attach an iron rod to the earth the upper end is negative and the lower end positive, and this principal is as true in the cavity of a tooth as it is in the atmosphere; every metal filling passing from the crown of a tooth to the cervical border is polarized when the mouth is opened for breathing or changes of temperature. When exposed, the portion covered by food or moisture is positive. Do not understand me as saying one end of the gold filling is positive and the other negative; but one end is covered by food or other substances, and by decomposition at the cervical edges becomes polarized, and this coming in contact, an acid is the result, and it readily deposits by galvanic action upon the surface where this takes place, whatever metals may be in the mouth where this decomposition is going on. There are all the means of decomposition present by this process. This occurs in fillings composed of one metal and in fillings of tin covered with gold. The tin becomes positive and the gold negative, and it thus forms an oxide or sulphide of silver, and this is received into the porous or softened dentine, which greatly retards decomposition of tissue.

Dr. W. D. Miller, of Berlin, does not agree with me upon the subject of polarity, but did agree in a paper presented by him last year, that the amalgams hinder decomposition better than gold. The amalgam in contact with gold is acted upon in a greater degree, and by this increased action is rendered negative, much more than if the gold is not present, and destroys low forms of life without question. The application I make is that by reason of decomposition, the gold is very apt to receive any metal held in solution, the gold being the negative element.

To my mind there is no more mystery in the discoloration of gold than in the deposition of one metal upon another by the electro-galvanic process being the same in the one case as in the other.—*Odontographic Journal.*

